# Ion Mobility Spectrometry as a Potential Tool for Flavor Control in Chocolate Manufacture

**DOI:** 10.3390/foods8100460

**Published:** 2019-10-09

**Authors:** Carolin Schmidt, Doris Jaros, Harald Rohm

**Affiliations:** Chair of Food Engineering, Technische Universität Dresden, 01062 Dresden, Germany; doris.jaros@tu-dresden.de (D.J.); harald.rohm@tu-dresden.de (H.R.)

**Keywords:** ion mobility spectrometry, cocoa processing, conching, flavor volatiles

## Abstract

Chocolate has a complex flavor profile composed of more than 600 volatile compounds that mainly arise from the thermo-mechanical treatment during roasting and conching. The aim of this study was to evaluate the applicability of ion mobility spectrometry (IMS), as a real-time method for process monitoring in chocolate manufacture. It is evident from the ion mobility (IM) fingerprint spectra that individual processing steps affect the signal intensities at particular drift time regions. The analysis of individual IM spectra by principal component analysis (PCA) revealed that it is possible to distinguish with respect to conching temperature and time. PCA also allowed identifying those parts of the IM spectra that were predominantly affected by the respective treatment. It was, on the basis of the IM flavor fingerprints and subsequent PCA, possible to distinguish between the different states of processing of bulk cocoa. The results of the study imply that, using appropriate post-data treatment, IMS could be used for process control in cocoa processing.

## 1. Introduction

Chocolate products show typical flavor profiles that are associated to the mixture of chemical compounds arising (a) from the primary cocoa aroma (cultivar and provenience) and (b) from secondary flavors that result from the complex physico-chemical conversions during fermentation, roasting, and conching [1,2]. In this context, more than 600 volatile compounds of several chemical substance classes including aldehydes, ketones, esters, alcohols, pyrazines, and others have been identified [1,3]. Generally, cocoa processing is characterized by the formation, degradation or evaporation of different compounds, and the increase or decrease of key substances content may be used for quality monitoring [3,4,5].

Cocoa processing starts with a sequence of biochemical reactions during fermentation where aroma precursors such as reducing sugars, free amino acids, and short-chain peptides are formed. In addition, non-volatile and volatile flavor compounds are produced (for instance, alcohols, short-chain organic acids and esters) [6], and the potential for the microbial synthesis of methylpyrazines [7] and for the formation of aldehydes [6,8] was also confirmed. The central step of flavor formation is roasting which, at temperatures above 100 °C, triggers chemical reactions of the cocoa ingredients. Predominantly, the Maillard reaction and Strecker degradation of Maillard reaction products cause a significant increase of acids, aldehydes, ketones and methylpyrazines, mainly trimethylpyrazine and tetramethylpyrazine [3,6,8,9,10]. The last processing step relevant with respect to flavor is conching, which significantly contributes to the formation of the final chocolate flavor. In addition, residual volatile acids such as acetic acid, alcohols (e.g., linalool and 2-phenylethanol), and other off-flavors (e.g., ketones and aldehydes) are removed [1,3,4,11].

Gas chromatography-mass spectrometry (GC-MS), possibly coupled with headspace solid phase microextraction (HS-SPME), is a sophisticated and time-consuming method that is, after adequate sample preparation, used by default for the identification of single flavor compounds in cocoa and chocolate [1,12,13,14]. In contrast, ion mobility spectrometry (IMS) is a physical method that allows a mobility-based separation of volatile and semi-volatile compounds in a weak electric field at ambient pressure [15]. IMS was, among others, initially used for the real-time determination of traces of explosive agents and drugs at airport safety checks. In the last fifteen years a wide range of new IMS applications has been developed, for instance in medical and clinical diagnostics, in the pharmaceutical sector to verify cleaning processes, and for emission measurements in the environment [16]. As a fast, sensitive and cost-efficient method, which is applicable mobile and in-line, IMS is predestined for food quality and safety control [17]. Volatile biogenic amines were successfully determined in meat and fish [18,19], and Garrido-Delgado et al. [20] showed that it is possible to distinguish wines or olive oils using IMS coupled to a gas chromatography (GC) column. Specially equipped IMS systems were used to detect mycotoxin traces in pistachios and licorice roots [21,22], and for differentiation of spice blends and the detection of adulteration [23].

The central part of an ion mobility (IM) spectrometer is the drift tube that is split by an electric shutter grid into an ionization room and a drift room. By applying high voltage an electric field is generated [15,24]. A carrier gas is used to introduce the gaseous sample into the drift tube where it is ionized using an appropriate ionization source (e.g., ultraviolet (UV) light, radioactive radiation source). When a radioactive source is used, for instance tritium, the carrier gas itself is ionized. A sequence of ion-molecule-reactions with nitrogen and hydrogen leads to the formation of hydrated hydronium ions, which are denoted as reactant ions and occur as reactant ion peak (RIP) in the IM spectrum. Subsequently, the reactant ions transfer their charge to the analyte molecules by proton transfer reactions which lead to a decrease of the RIP intensity [24]. After ionization the shutter grid is periodically opened so that analyte ions can enter the drift room where they are accelerated in the electric field in the direction of the Faraday plate that serves as a detector. The drift gas that flows in opposite directions prevents the neutral analyte molecules to enter the drift room. In addition, the drift gas molecules collide with the analyte ions and slow them down depending on size, form and charge so that they receive a characteristic drift speed and ideally reach the detector successively [17]. The time the analyte ions need to move from the shutter grid to the detector is the drift time, and the signal of the detector recorded as a function of drift time represents the IM spectrum [24].

Another fast approach for flavor control during cocoa processing is to use an electronic nose by which, for instance, Tan and Kerr [25] monitored volatile compounds during refining and conching. Similar to IMS, an E-nose is not able to directly identify any specific compound and, additionally, the life time of the sensors is limited [26]. Rottiers et al. [27] discriminated cocoa liquors by an ultra-fast GC electronic nose. Such a coupled E-nose can identify several volatile compounds, but is much more complex in construction than an IMS.

The aim of this study was to investigate the potential of IMS without any additional upstream equipment for a fast and real-time fingerprint tracing of flavor development during cocoa processing. This is demonstrated using sample sets, comprising fermented raw cocoa beans, and roasted cocoa nibs on their way to final chocolate mass.

## 2. Materials and Methods

### 2.1. Cocoa and Chocolate Samples

For tracing flavor development during chocolate manufacture fermented cocoa beans, unroasted and roasted cocoa nibs, preground cocoa liquor, and finely ground and pre-treated cocoa liquor made from those beans were obtained from a German chocolate manufacturer. Approximately, 1 kg of each sample was split into five batches. Three of them were used for independent IMS measurements at different days within two weeks, and the remaining two were analyzed using HS-SPME-GC-MS. Dark chocolate mass was obtained from the same chocolate manufacturer for conching experiments. All samples were stored in tight plastic containers at 4 °C until analysis. Prior to measurements, fermented cocoa beans and nibs were comminuted using a mortar and a pestle.

### 2.2. Conching of Chocolate Mass

Chocolate masses were sheared and aerated in a DoCorder compounder (Brabender GmbH & Co. KG, Duisburg, Germany) to simulate conching in laboratory scale. 450 ± 1 g of dark chocolate mass was filled into the compounder cell and conching was realized at a fixed mixing speed of 150 rpm using a double z-kneader. Jacket temperature was maintained at 50 °C or 75 °C by a circulator. Conching was carried out in two independent batches for each temperature. Samples were taken after 10, 30, 60, 120, and 240 min and immediately transferred into glass vials for further duplicate analysis.

### 2.3. Headspace Solid Phase Micro-Extraction GC-MS Analysis

4.0 ± 0.1 g cocoa sample was weighed into a 20 mL glass vial, and closed with a PP cap with PTFE/silicon septum (Phenomenex GmbH, Aschaffenburg, Germany). The samples were then conditioned at 60 °C for 45 min in a water bath. Adsorption of volatiles was performed at 60 °C using an SPME unit equipped with a conditioned (15 min at 250 °C) 50/30 µm divinylbenzene/carboxen/PDMS stableflex fiber (Supelco Analytical, Bellefonte, PA, USA) which was inserted into the headspace above the sample for 45 min.

The cocoa volatiles were detected using a 7890 A GC equipped with a 7683 B injector and a HP-5ms column (Agilent 19,091 J-443: 30 m × 0.25 mm, 0.25 µm film thickness) coupled to an MSD 5975 C mass spectrometer (Agilent Technologies Deutschland GmbH, Waldbronn, Germany) with a source temperature of 230 °C. Measurements were carried out in 10:1 split mode at a helium flow of 1 mL/min. Based on Owusu et al. [28], oven temperature was increased from 40 °C (holding time 10 min) to 240 °C at 8 K/min, and final holding time was 5 min. After the fiber had been thermally desorbed in the GC injector for 5 min at 250 °C, the measurement was started. Identification of compounds was carried out by comparing mass spectra using NIST MS Search 2.0 (National Institute of Standards and Technology, Gaithersburg, MD, USA) and PBM Quick Search (Agilent Technologies, Inc., Santa Clara, CA, USA). Only substance peaks that were in accordance with more than 70% of the libraries data were evaluated. For comparison purpose, the absolute peak areas were used.

### 2.4. Ion Mobility Spectrometry

Analyses were carried out at ambient pressure using a SMELLMASTER^®^ IM spectrometer controlled by the IMS Monitor software 2.1.1.0 (IfU GmbH Privates Institut für Analytik, Frankenberg, Germany). The cylindrical drift tube of the instrument is 50 mm long and has a diameter of 10 mm. The IMS operated at an electrical field of 400 V/cm, corresponding to a total voltage of 2 kV. After ionization realized by β^−^ -radiation (^3^H, 50 mBq), the shutter grid was periodically opened for 60 µs. Measuring conditions, e.g., measuring mode, detector temperature, sample temperature, sample weight, were optimized in preliminary studies.

There was 2.0 ± 0.1 g of cocoa or chocolate weighed into a 20 mL glass vial that was closed with a PP cap with PTFE/silicon septum and conditioned at 60 °C for 60 min prior to and during measurement. Glass vials were connected to the sample inlet system (for detailed information on the measurement setup see Figure S1, Tzschoppe et al. [29]) by simultaneously piercing two stainless steel syringe needles (neoLab Migge Laborbedarfs-Vertriebs GmbH, Heidelberg, Germany) through the septum. After 5 min of pre-conditioning valve 1 of the sample inlet system was opened and the headspace of the vial was purged with carrier gas (compressed air, 1 L/min, dew point <−40 °C ensured by an ATK-APN 10+ ET-C adsorption dryer, dt druckluft-technik GmbH, Kirchheim, Germany) so that the volatile flavor compounds were transferred into the IMS. The sample inlet valve was manually adjusted to ensure that the RIP was 30–40% of its initial height of approximately 5.0 and was, in this study, therefore adjusted to 2.0. Measurements were carried out in positive mode at a detector temperature of 80 °C and a valve temperature of 50 °C. Drift gas flow rate was 400 mL/min, and sample inlet flow rate was 40 mL/min.

Drift time of the individual peaks was normalized to the drift time of the RIP at standard operation temperature of 80 °C and atmospheric pressure (101.23 hPa). Relative drift time t_D_ is therefore dimensionless. For each sample, the spectra were recorded after a 20 s equilibration interval for 2 min. During operation, an averaged spectrum consisting of 10 individual spectra was sent to the software every second. Every two seconds, these spectra were averaged again and stored by the system. Peak areas from the IM spectra of bulk cocoa were determined using OriginPro 8G SR2 (OriginLab Corperation, Northampton, MA, USA).

Immediately before and after measurement the IMS was purged with compressed air until the initial spectrum was restored. While purging, valve 1 in the sample inlet system was completely open.

### 2.5. Statistical Analysis

Principle component analysis (PCA) of the entire IM spectra in a t_D_ range from 1.03 to 1.36 was carried out using Unscrambler 10.3 (CAMO Software AS, Oslo, Norway). For the sake of clarity, loadings with endpoints in the area −0.12 ≤ PC1, PC2 ≤ 0.12 are not shown. Correlation analysis (*n* = 5) was done using areas of peaks from IM spectra and absolute peak areas of selected flavor compounds determined by HS-SPME-GC-MS. 

## 3. Results and Discussion

### 3.1. Ion Mobility Spectra of Chocolate Mass during Conching

**Figure 1:**
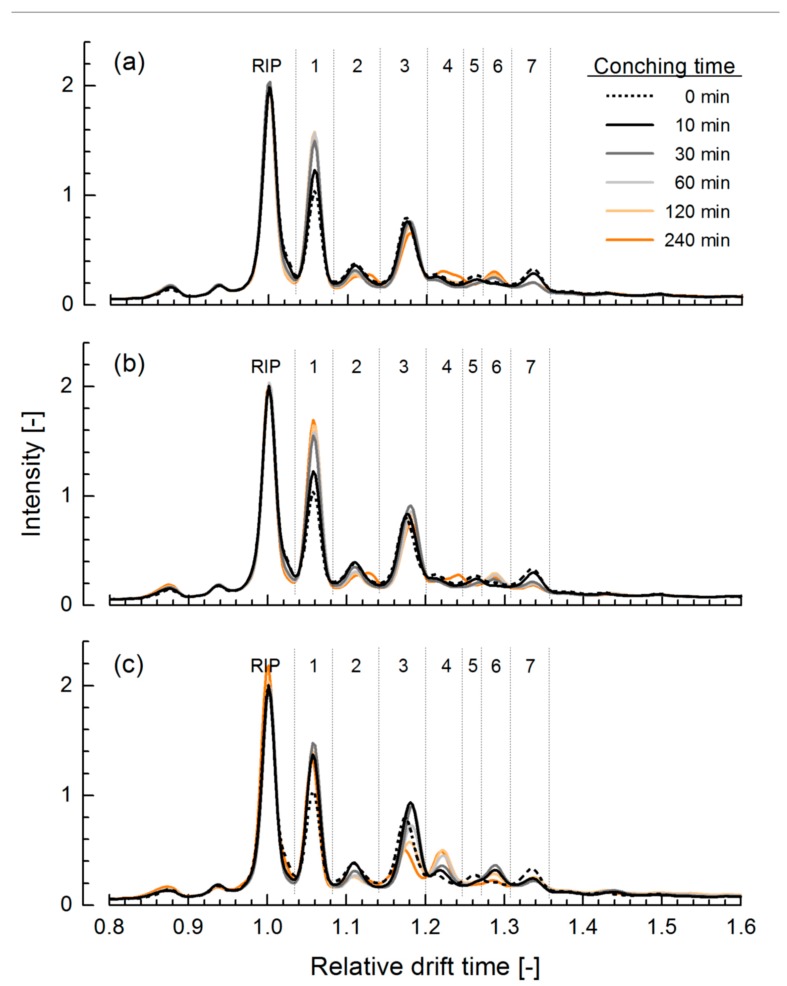
Ion mobility (IM) spectra of dark chocolate mass, conched in duplicate at 50 °C (**a**,**b**) and 75 °C ((**c**), only one trial shown) as a function of conching time. Dashed lines and the indicator “0 min” refer to the chocolate mass before conching. RIP: reactant ion peak.

Figure 1 shows the IM fingerprints of chocolate mass that was conched at two different temperatures in two independent duplicate batches. The spectra of the duplicate batches were similar. The fingerprints show seven characteristic peaks after the RIP, and the water and ammonium peak in front of the RIP. In all trials, the RIP was adjusted to a relative intensity of approx. 2.0 by regulating valve 1 in the sample inlet system (see Figure S1). The intra- und inter-day performance of the method is, using cocoa liquor, exemplary shown in Figure S2. Peak 1 at t_D_ ≈ 1.06 had the highest intensity in all samples. At 50 °C (Figure 1a,b) the height of this peak increased until 30 min of conching; afterwards, peak intensity remained constant. At a conching temperature of 75 °C (Figure 1c) this peak already reached its maximum height after 10 min. The intensity of peak 2 (t_D_ = 1.11) slightly decreased with conching time, but was not affected by conching temperature. The latter had, however, a pronounced influence on both intensity and position of peak 3 (t_D_ ≈ 1.18). At 50 °C, the peak maximum decreased with conching time and was slightly shifted towards a higher drift time. At 75 °C, peak intensity firstly increased from 0.78 to 0.94 but, after 30 min of conching, decreased to 0.51. The peak maximum was also shifted along the *x*-axis, at the beginning of conching slightly to a higher t_D_ and, after 60 min conching, slowly back to its original position. The maximum of peak 4 (t_D_ ≈ 1.22) increased and was slightly shifted to the right with increasing conching time, with the peak height increase being more pronounced at higher conching temperature. The next part of the IM spectra showed two small peaks, peak 5 at t_D_ ≈ 1.26 and peak 6 at t_D_ ≈ 1.29. Chocolate mass before conching, denoted as “0 min sample” in Figure 1, showed only peak 5. With increasing conching time the height of peak 5 decreased and that of peak 6 slightly increased; after 30 min of conching, peak 5 was no longer visible. At 75 °C, peak 5 already disappeared after 10 min. The height of peak 7 (t_D_ ≈ 1.33) decreased during conching and remained constant after 30 min at 50 °C, and after 10 min at 75 °C. 

It is known from the literature [10] that during conching, water vapor volatile compounds are removed from the chocolate mass. Therefore, it is highly likely that decreases of peak height in the IM spectra are an indicator for such a removal. Consequently, increases in peak height presumably refer to flavor compounds that become more prominent because of redistribution or the removal of other compounds. It might also be possible that increasing peaks indicate the presence of pyrazines. Literature statements referring to pyrazine concentration changes during conching are, however, contradictory. Albak and Tekin [30] and Perego et al. [14] determined a reduction of several pyrazines, whereas Counet et al. [4] and Ascrizzi et al. [31] showed a concentration increase.

**Figure 2:**
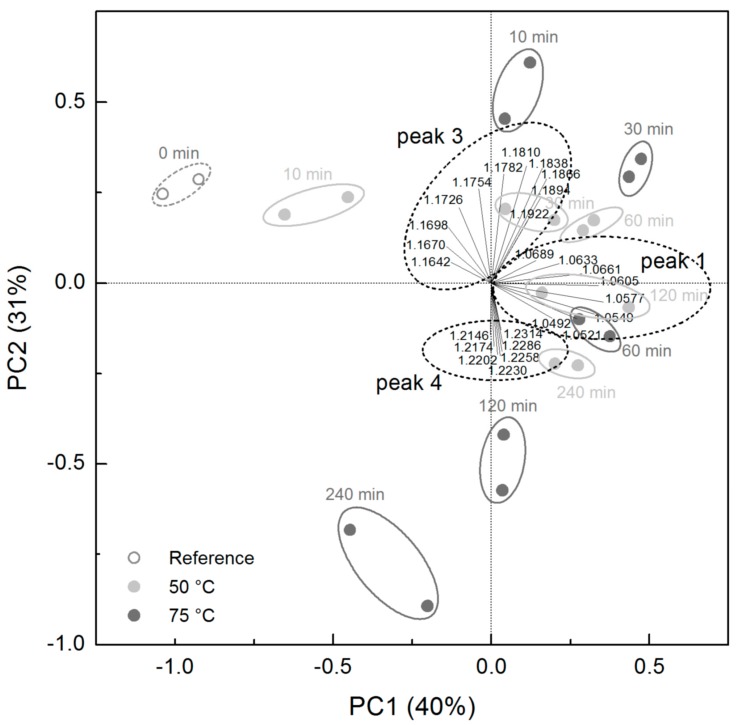
Principal component analysis of IM spectra of dark chocolate mass after different times of conching at 50 °C or 75 °C. Reference is chocolate mass before conching. Dotted line ellipses indicate groups of relative drift times identified at the respective peaks.

After subjecting the entire set of IM fingerprints to PCA, the conching times at the respective temperature appear as scores in the biplot (Figure 2). Each sample is positioned close to its independent duplicate, and the first two principal components (PC) already explain 71% of the total variance. Loadings further away from the point of origin describe those peaks that are more pronounced and that differ in peak height to a larger extent. The three groups of loadings shown in the biplot refer to the drift times of peak 1 (t_D_ ≈ 1.06), peak 3 (t_D_ ≈ 1.18) and peak 4 (t_D_ ≈ 1.22). Samples that were conched at 50 °C can be distinguished mainly by PC1, and only the samples conched for 120 and 240 min differed from the remaining samples in the direction of PC2. This finally indicates that the differences between the fingerprints of the samples that were conched at 50 °C during the first two hours can be described by the peak at t_D_ ≈ 1.06 (peak 1). The differences between the 120 min and 240 min conched samples and all other ones conched at 50 °C can be mainly ascribed to peaks 3 and 4. All samples that were conched at 75 °C differed both in PC1 and in PC2, meaning that all peaks reflect changes in sample composition. Peak 3 with a maximum height at t_D_ ≈ 1.16–1.18 is spreading equally strong over both PC in the biplot. Consequently, and independent of conching temperature, samples that are located in the left part of the biplot showed a maximum of peak 3 at lower t_D_. Samples with higher PC2 scores in the biplot had higher heights in peak 3. The loadings of peak 4 point towards negative values of PC2 in the biplot. This means that samples with the lowest PC2 scores had the highest intensities of peak 4. Samples that were conched at 50 °C for 120 and 240 min are located close to those that were conched at 75 °C for 60 min. This indicates that the removal and redistribution of volatile compounds that is a function of conching time and that takes place much faster when conching temperature is higher [1,10] can also be identified by IMS measurements.

### 3.2. Ion Mobility Spectra of Bulk Cocoa Samples during Processing

**Figure 3:**
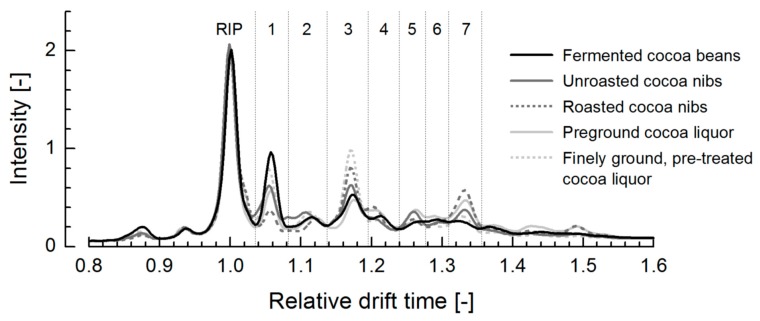
IM spectra of bulk cocoa (*n* = 3).

Bulk cocoa samples from several processing states showed IM fingerprints with seven peaks after the RIP at nearly the same t_D_ as in the conching spectra (Figure 3). Depending on the processing state, especially peaks 1, 3 and 7 varied in their height and peak areas. The height of peak 1 (t_D_ ≈ 1.05) decreased strongly during processing, achieved its lowest intensity after roasting and then increased again for the grinded and pre-treated cocoa liquor. Maximum of peak 3 (t_D_ ≈ 1.17) slightly increased during crushing and roasting, then decreased during pre-grinding and finally reached its highest intensity after grinding and pre-treatment. Crushing and roasting led to an increase of peak 7 (t_D_ ≈ 1.33), and the intensity of this peak decreased through grinding and pre-treatment.

**Figure 4:**
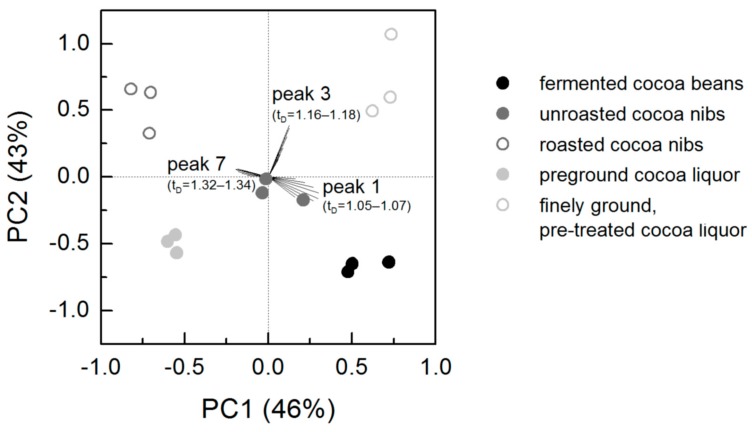
Principal component analysis of IM spectra of bulk cocoa (*n* = 3).

All single measurements were subjected to PCA where PC1 and PC2 explain 89% of the total variance (Figure 4). The loadings of peaks 1, 3 and 7 with the indicated drift times were the most defined ones, whereas the loadings of the other four peaks were located close to the point of origin and are therefore not shown. Scores of samples that represent the same processing state appear as groups in the biplot, meaning that the different processing states can be clearly separated from each other by IMS. The unroasted nibs are located very close to the point of origin and therefore exhibited a medium intensity at peaks 1, 3 and 7. The scores of the roasted nibs and the fermented cocoa beans are located in the 2nd and the 4th quadrant of the biplot in the direction of the loadings from peak 7 and peak 1. Consequently, roasted nibs and fermented cocoa beans can be distinguished from unroasted nibs mainly by peak 1 and peak 7 rather than by peak 3. Pre-ground cocoa liquor and finely ground, pre-treated cocoa liquor differed especially in peak 3 from the other samples, the latter being the only sample in the 1st quadrant of the biplot with the highest intensity of peak 3.

### 3.3. Correlation of Ion Mobility Spectrometry and HS-SPME-GC-MS Analysis

Peak areas of the seven peaks in the IM spectra were integrated, and the correlation with the peak areas of selected flavor compounds (alcohols, ketons, pyrazines esters, and aldehyds) determined by HS-SPME-GC-MS (absolute peak areas of all compounds analyzed are shown in Table S1) was investigated. The compounds that were selected were those nine volatiles that were detected in at least four of the five processing states, and that had the largest peak areas in the chromatograms and, additionally, nonanal because of high correlation.

**Figure 5:**
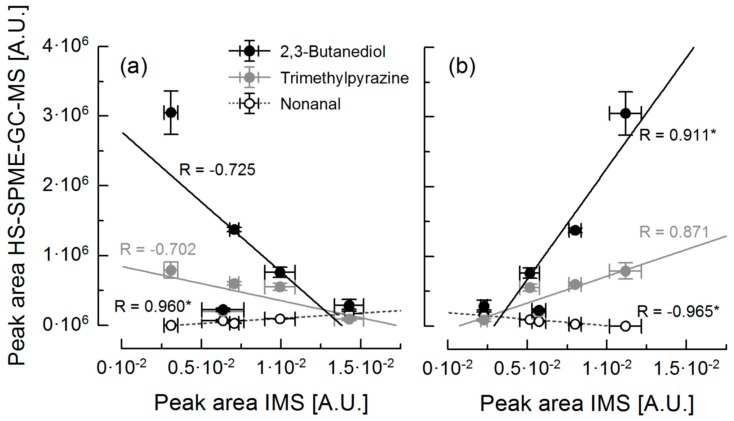
Correlation analysis of peak areas of 2,3-butanediol, trimethylpyrazine and nonanal determined by HS-SPME-GC-MS (*n* = 2) and peak areas of peak 1 (**a**) and peak 7 (**b**) of IM spectra (*n* = 3) of bulk cocoa samples. * marked correlation coefficients are significant (*p* < 0.05). [A.U.]: arbitrary unit. IMS: ion mobility spectrometry

Table 1 gives an overview of the correlation coefficients between the areas of the seven IMS peaks and the peak areas of selected flavor compounds that were identified by HS-SPME-GC-MS. For 2,3-butanediol, trimethylpyrazine and nonanal, the correlations with the areas of peaks 1 and 7 are shown in Figure 5. There are peaks whose area correlated significantly with the peak area of different compounds, for example peak 3 and peak 7. There are also flavor compounds whose peak areas correlated significantly with the area of several peaks, see for instance 2,3-butanediol or nonanal. This proves that one single peak in IM fingerprint may result from a combination of a few compounds, and that one single compound can raise or decline several peaks. This is exemplary demonstrated by the IM spectra of trimethlypyrazine, linalool and nonanal, shown in Figure S3. The presence of two peaks in the IM spectrum of pure hexanal was also described by Tzschoppe et al. [29]. Two peaks can be considered as an indicator for the existence of monomers and dimers of the respective compound. The presence of dimers is usually caused by a high concentration of the respective molecules and leads to additional peaks in the spectrum [24,26,32]. In our study, the concentration of some compounds correlated positively, and that of others correlated negatively with the peak areas from the IM spectra. A negative correlation could be due to the fact that, in the presence of monomer and dimer peaks, the monomer peak decreases while the dimer peak is increasing when the concentration of the compound increases [28]. The decrease of a peak despite an increase in the concentration of this compound could also be attributed to the removal of another compound that was represented in the same peak in the IM spectra.

## 4. Conclusions

The results of this study demonstrate the potential of using IMS for real-time process monitoring in chocolate manufacture. It was, for example, possible to clearly distinguish chocolate masses that were conched at different temperature for different periods of time. This was realized by post-measurement treatment of the IM fingerprint spectra by PCA that identified t_D_ = 1.06, 1.18 and 1.22 as being most significant. Additionally, it was possible to distinguish between different processing states of bulk cocoa. Some peak areas of the IM spectra correlated significantly with absolute peak areas of selected flavor compounds analyzed by HS-SPME-GC-MS. For a proper localization of such compounds in the IM fingerprint spectra, measurements with pure flavor substances are necessary.

## Figures and Tables

**Table 1:** Correlation coefficients R between peak areas of selected compounds determined by HS-SPME-GC-MS and peak areas of peaks 1 to 7 from IM fingerprint spectra of bulk cocoa samples (*n* = 5).

Compound	R_peak 1_	R_peak 2_	R_peak 3_	R_peak 4_	R_peak 5_	R_peak 6_	R_peak 7_
2,3-Butanediol	−0.725	−0.556	0.080	0.904 *	−0.094	−0.232	0.911 *
1-Butanol-3-methyl-acetate	−0.006	−0.152	−0.983 *	0.292	0.540	0.776	0.000
Benzaldehyde	−0.323	0.266	−0.146	−0.338	0.665	−0.244	−0.034
Trimethylpyrazine	−0.702	−0.090	0.331	0.654	0.057	−0.479	0.871
Acetophenone	0.166	−0.513	−0.944 *	0.310	0.154	0.875	−0.152
Tetramethypyrazine	−0.619	0.363	0.543	0.250	0.237	−0.713	0.681
Linalool	−0.156	0.677	0.041	−0.610	0.718	−0.355	−0.190
Nonanal	0.960 *	0.065	−0.018	−0.665	−0.522	0.438	−0.965 *
Phenethyl alcohol	−0.483	0.227	−0.004	0.502	0.354	−0.153	0.658
Phenethyl acetate	−0.338	0.858	0.349	−0.280	0.621	−0.577	0.234

* Marked correlation coefficients are significant (*p* < 0.05).

## Supplementary Materials

The following are available online at https://www.mdpi.com/2304-8158/8/10/460/s1, Figure S1: IMS Sample inlet system; Figure S2: IM spectra of cocoa liquor (used for testing purposes) that was measured repeatedly on several days. Peak areas in table are mean value ± standard deviation (*n* = 6) of the seven peaks in IM spectra; Figure S3: IM spectra of pure flavor compounds usually found on cocoa and chocolate. Intensity axis: RIP intensity for each compound is 2.0; Table S1: Absolute peak areas of flavor compounds determined by HS-SPME-GC-MS (*n* = 2).
