# Supplementary material for: Ion Mobility Spectrometry as a Potential Tool for Flavor Control in Chocolate Manufacture
Source: Foods. 2019 Oct 9;8(10):460. doi: 10.3390/foods8100460 (PMC6836128; doi:10.3390/foods8100460)
Supplement: Supplementary file 1 [file foods-08-00460-s001.pdf]

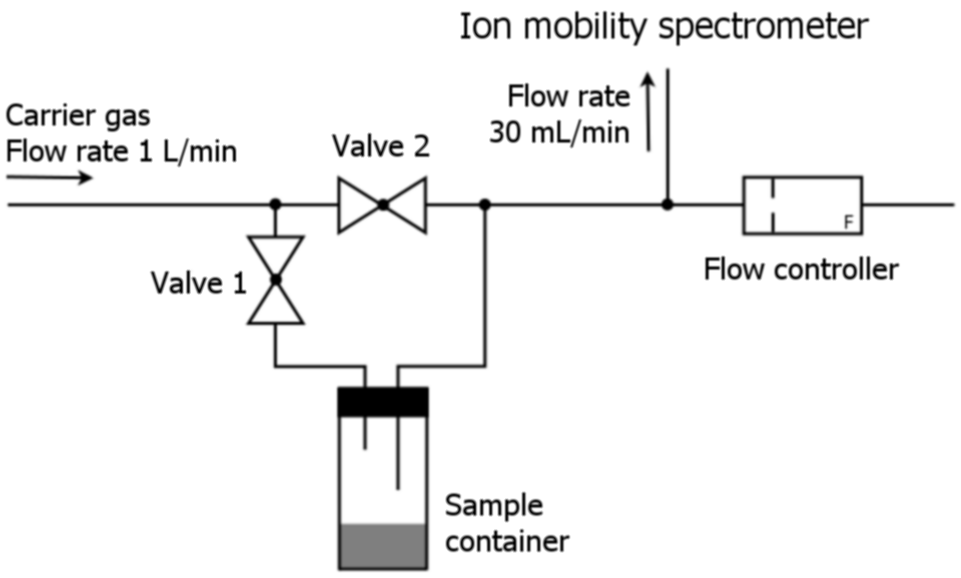

Figure S1. IMS sample inlet system. From M. Tzschoppe et al., Using ion mobility spectrometry for screening the autoxidation of peanuts, Food Control 2016, 64, 17–21, with permission from Elsevier.

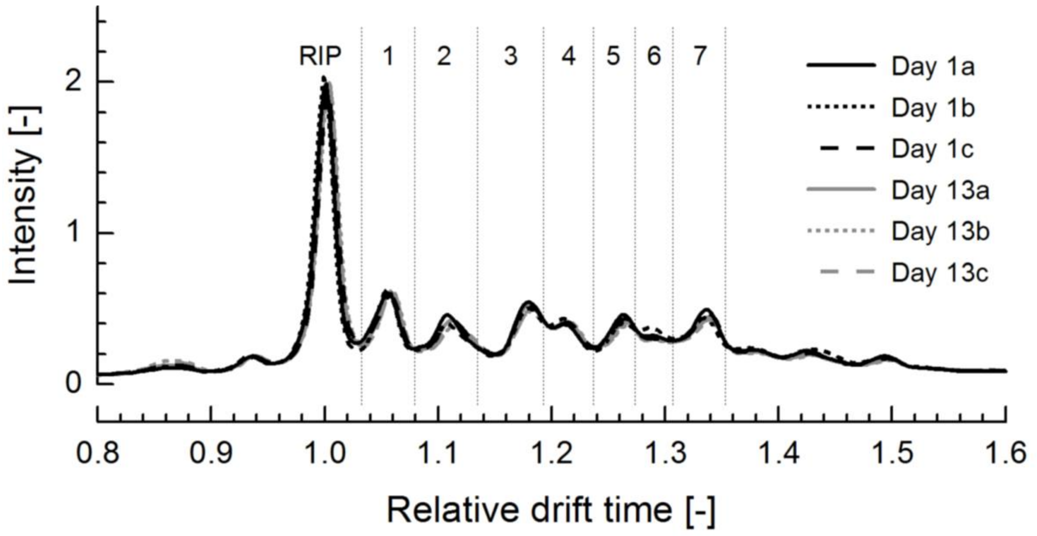

	Peak 1	Peak 2	Peak 3	Peak 4	Peak 5	Peak 6	Peak 7
Peak area [10^{-2} · A.U.]	0.76 ± 0.04	0.57 ± 0.04	0.87 ± 0.07	0.45 ± 0.06	0.48 ± 0.06	0.17 ± 0.04	0.68 ± 0.06
RSD [%]	5.7	6.3	8.5	13.3	11.7	23.5	8.5

Figure S2. IM spectra of cocoa liquor (used for testing purposes) that was measured repeatedly on several days. Peak areas in table are mean value ± standard deviation (n = 6) of the seven peaks in IM spectra.

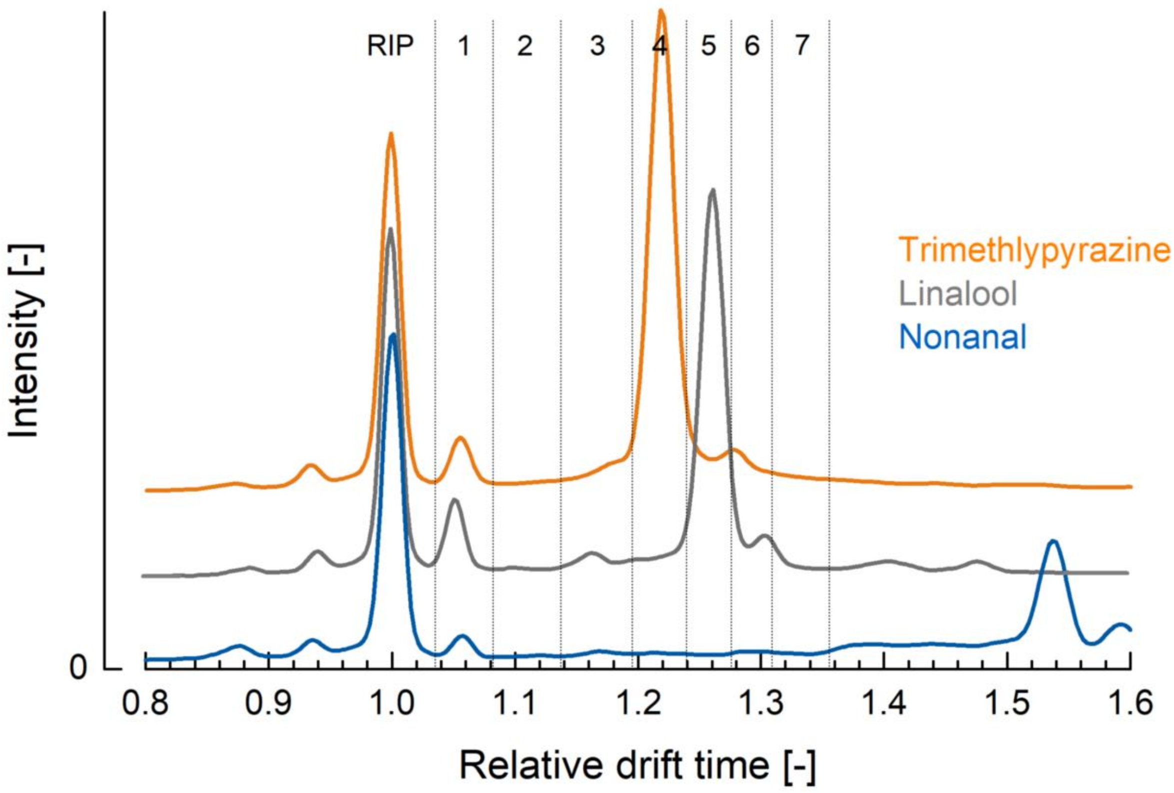

Figure S3. IM spectra of pure flavor compounds usually found on cocoa and chocolate. Intensity axis: RIP intensity for each compound is 2.0.

Table S1. Absolute peak areas of flavor compounds determined by HS-SPME-GC-MS (n = 2).

Compound	Absolute peak area [10^4 · A.U.]				
	**Fermented cocoa beans**	**Unroasted cocoa nibs**	**Roasted cocoa nibs**	**Preground cocoa liquor**	**Finely ground, pretreated cocoa liquor**
Acetoin	14.6	21.1	-	-	-
3-Methylbutan-1-ol	44.5	12.6	-	-	-
2-Methylpropanoic acid	42.2	-	-	-	-
2,3-Butanediol*	28.5	22.5	305.0	137.4	76.1
Pentyl acetate	43.2	40.0	-	-	-
1-Butanol-3-methyl-acetate*	64.1	61.3	37.7	89.1	-
1-Butanol-2-methyl-acetate	17.9	27.8	-	-	-
2-Heptanol	46.5	17.8	-	-	-
2,5-Dimethylpyrazine	-	-	36.6	26.0	12.4
2,3-Dimethylpyrazine	-	-	26.5	19.3	14.7
Benzaldehyde*	34.2	100.6	40.1	41.2	36.2
Beta-myrcene	39.6	17.6	8.7	9.0	6.9
2-Ethyl-6-methylpyrazine	-	-	3.1	3.1	2.9
Trimethylpyrazine*	8.8	19.5	79.0	59.9	55.1
Limonene	3.7	6.1	3.4	4.4	4.6
Phenylacetaldehyde	-	-	9.4	18.8	20.6
2-Isobutyl-4,5-dimethyl-3-oxazoline	-	-	78.3	-	-
1-(1H-Pyrrol-2-yl)ethanone	-	-	11.8	20.5	21.1
Acethophenone*	23.0	21.0	20.1	21.4	16.0
3-Ethyl-2,5-dimethylpyrazine	-	-	13.9	16.0	16.7
Tetramethylpyrazine*	77.4	181.2	281.2	276.8	313.9
2-Nonanone	15.0	29.6	17.7	21.1	19.5
Linalool*	21.6	35.1	20.7	25.9	26.7
Nonanal*	16.8	6.8	-	2.9	9.2
2-Isopropyl-5-methyl-2-hexenal	-	-	78.6	13.8	-
2-Phenylethanol*	71.1	87.8	181.5	257.3	169.1
Massoia lactone	-	-	56.0	127.7	65.1
2-Ethyl-3,5,6-trimethylpyrazine	-	4.5	11.7	11.1	12.6
Ethyl octanoate	7.4	4.2	5.0	7.9	6.9
2-Phenethyl acetate*	29.1	56.2	42.5	64.3	65.8
5,6-Dihydro-2H-pyran-2-one	-	-	-	11.0	19.2
2-Phenyl-2-butenal	-	8.1	11.0	21.4	20.9
Amyl benzoate	10.0	12.0	8.7	14.4	15.7
Caffeine	-	-	-	14.7	10.8

* Marked compounds are the selected volatiles for correlation analysis.
